# *CLEC11A* methylation is correlated to AML subtypes and cytogenetic risk factors but not patient demographics

**DOI:** 10.1371/journal.pone.0300477

**Published:** 2024-03-11

**Authors:** Allyson J. Swanson, Victor J. Rogowski, Jacob A. Bishop, Dylan M. Walker, Gina M. Roxas, Stacey L. Raimondi

**Affiliations:** 1 Department of Biology, Elmhurst University, Elmhurst, Illinois, United States of America; 2 Department of Chemistry and Biochemistry, Elmhurst University, Elmhurst, Illinois, United States of America; 3 Environmental Studies Program, Elmhurst University, Elmhurst, Illinois, United States of America; Bursa Ali Osman Sonmez Oncology Hospital, TURKEY

## Abstract

Acute myeloid leukemia (AML) is an aggressive and lethal cancer of the blood, which leads to the death of over 11,000 patients in the United States each year. Research on identifying, characterizing, and treating AML is crucial in the fight against this deadly disease. Recent studies have examined the role of *CLEC11A* in cancer, including AML. However, there have been conflicting reports related to tumor progression and survival. Because survival is based on a variety of factors, including classification of the tumor, genetic risk factors, and demographics, it is imperative that we determine what role *CLEC11A* may have in cancer survival. Therefore, utilizing data from the Genomic Data Commons, we analyzed *CLEC11A* methylation in 108 AML patients compared to FAB classification, cytogenetic risk factors, age, race, and gender. Our results show statistically significant correlations between methylation of *CLEC11A* and FAB classification as well as poor genetic risk factors. However, no difference was observed in *CLEC11A* methylation when compared to demographic data. Our results, matched with a known biological function of *CLEC11A* in early hematopoiesis, indicate that *CLEC11A* may be an important marker for AML diagnosis and prognosis and provide relevant data in the ongoing search for novel therapeutics to improve AML survival.

## Introduction

Acute myeloid leukemia (AML) is an aggressive and fast-acting cancer in which the bone marrow produces abnormal white blood cells, red blood cells, or platelets. AML is a highly heterogeneous disease that makes up almost a third of all leukemia diagnoses [1]. Newly diagnosed patients have an average survival of approximately 17 weeks if untreated [2]. Even with treatment, patients’ 5-year relative survival rate is 30.5 percent [3]. Risk factors for AML include gender (male), increased age, smoking, history of chemotherapy or radiation therapy, treatment for acute lymphoblastic leukemia as a child, exposure to the chemical benzene, and a history of blood disorders such as myelodysplastic syndrome [4]. AML staging uses the French American British (FAB) morphology staging system, which categorizes AML into subtypes M0 through M7. Unlike other cancers, there is no progression between AML stages. The subtypes, despite their numerical value, have no relationship to disease progression; however, subtype M0 is considered the worst diagnosis as cells are undifferentiated and difficult to treat. FAB staging and diagnosis are based on the amount of healthy blood cells within the body, the number and size of the leukemia cells, and the chromosome changes within the leukemia cells [4, 5]. Treatments for AML include high and low dose chemotherapy, targeted drug therapies, and stem cell transfers [4]. There are also a variety of experimental drug therapies currently in clinical trials [6]. However, poor survival rates and limited treatment options indicate the need for additional studies into the molecular mechanisms which may cause this disease.

*C-type lectin domain containing 11A* (*CLEC11A)*, also known as *P47*, *SCGF*, *LSLCL*, and *CLECSF3*, is located on chromosome 19 and encodes a protein that acts as a growth factor for stem cells and bone marrow cells [7–9]. CLEC11A is also necessary for osteogenesis, the process of creating new bone cells, to help repair injured bones. It functions through the ERK signaling pathway, which is responsible for regulating cell growth, development, and division [10, 11]. CLEC11A-expressing lung cancer cells have been shown to promote the growth of tumors in mice while inhibition of *CLEC11A* expression prevented tumor growth [12]. Interestingly, Yin and colleagues recently reported that increased methylation of *CLEC11A*, corresponding to decreased gene and protein expression, led to poor survival in AML patients [13]. These conflicting reports indicate that further studies are needed to elucidate the role of CLEC11A in cancer, with specific attention to the role of methylation.

DNA methylation is a normal epigenetic mechanism that regulates the expression of genes either by recruiting proteins involved in gene repression or inhibiting the binding of transcription factors [14]. Hypermethylation occurs when many methyl groups are bonded to cytosine bases on DNA, leading to a loss of gene expression due to the inability of transcription factors to bind to DNA. Conversely, when DNA is hypomethylated, it is free to be transcribed and translated into protein. Most methylation of DNA takes place at CpG sites, cytosine nucleotides that precede guanine nucleotides, usually within a CpG island in the promoter region of a gene [14].

As described above, previous conflicting studies have shown that increased CLEC11A expression is correlated with tumor progression in lung cancer and survival in AML [12, 13]. Survival rates incorporate many different factors: the age of patients, type of chemotherapy, stage of cancer, race, gender, and time from diagnosis to treatment [4]. While previous studies showed a relationship between *CLEC11A* and survival, these other factors were not considered specific to methylation of *CLEC11A* [12, 13]. Therefore, the goal of this study was to examine the relationship between *CLEC11A* methylation, staging, genetic risk factors, and demographic data to determine whether *CLEC11A* can be used as a prognostic factor in AML patients.

## Materials and methods

Gene methylation and clinical data for 122 AML patients were obtained from the GDC Data Portal (https://portal.gdc.cancer.gov/repository) in August 2022 as previously described [15, 16]. All data on GDC are deidentified with no way to identify patients outside of their unique case ID number. Within the data portal, the methylation levels of the three different components of *CLEC11A* (North Shore, Island, South Shore) of each patient were obtained by matching *CLEC11A* methylation and clinical data using the unique patient ID number [15].

Averages for each methylation level per clinical subtype, as well as for the patients’ cytogenetic risk factors, race, gender, and age were calculated. Total methylation was calculated as the sum of North Shore, Island, and South Shore methylation levels. Age divisions were based on the median age present in the 108 patient samples available on GDC. Statistical significance was calculated using an ANOVA with Tukey HSD analysis of all methylation data in relation to FAB morphology staging. A Kruskal Wallis with Dunn’s multiple comparisons and Bonferroni correction was utilized to determine statistical significance for cytogenetic risk factors. Statistical significance of demographic data was calculated with a Mann-Whitney Rank Sum test.

## Results

### *CLEC11A* total and north shore methylation levels are significantly altered depending on AML FAB classifications

**Fig 1:**
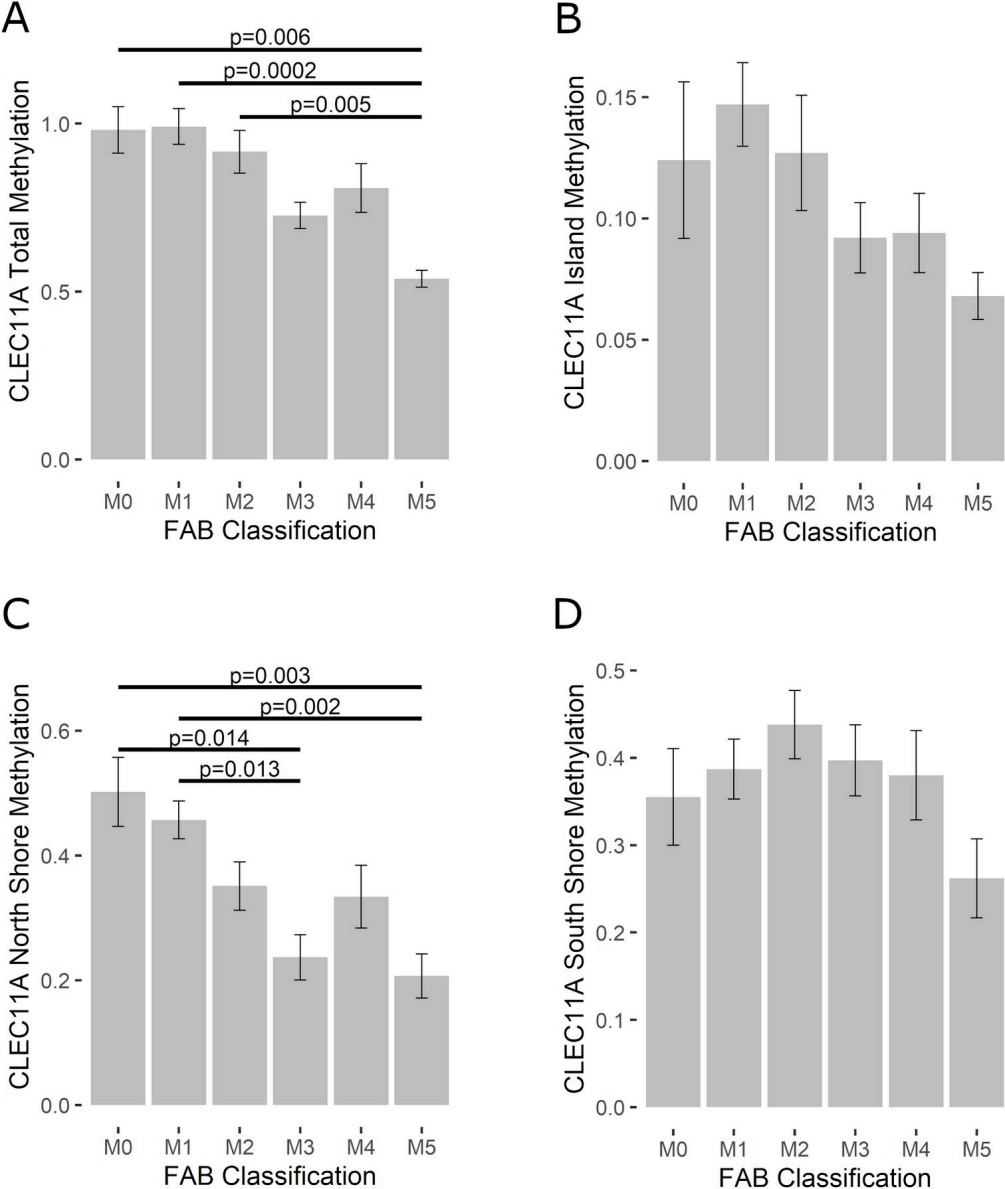
Methylation of *CLEC11A* across FAB classifications. Average methylation of the *CLEC11A* gene was analyzed based on AML subtype. Total methylation (A; *p* = 0.00002142), as well as methylation at the CpG island (B; *p* = 0.1232), North Shore (C; *p* = 0.00009314), and South Shore (D; *p =* 0.2556) are shown. Statistical significance was determined utilizing a one-way ANOVA with Tukey HSD. Significance between individual subtypes is indicated on graphs as appropriate.

To elucidate the role of *CLEC11A* in AML survival, we evaluated methylation levels of *CLEC11A* in patients diagnosed with AML based on their FAB morphology classification (M0 n = 12; M1 n = 36; M2 n = 27; M3 n = 11; M4 n = 22; M5 n = 12). Total methylation (North Shore, Island, and South Shore combined) was analyzed as well as methylation at each individual location on the *CLEC11A* promoter. ANOVA analysis indicated statistically significant results for both total methylation (*p* = 0.00002142) and methylation at the North Shore (*p* = 0.00009314; Fig 1). Pair-wise Tukey analysis resulted in additional statistically significant findings between the following groups in the total methylation analysis: M0 and M5 (*p* = 0.006), M1 and M5 (*p* = 0.0002), and M2 and M5 (*p* = 0.005; Fig 1A). Tukey analysis of methylation at the North Shore of *CLEC11A* compared to FAB classifications yielded significance from M0 and M3 (*p* = 0.014), M1 and M3 (*p* = 0.013), M0 and M5 (*p* = 0.003), and M3 and M5 (*p* = 0.002; Fig 1C). Finally, methylation levels at the Island (*p* = 0.1232; Fig 1B) and South Shore *(p =* 0.2556; Fig 1D) of *CLEC11A* showed no significant differences between FAB classifications.

### *CLEC11A* methylation correlates with cytogenetic risk factors

**Fig 2:**
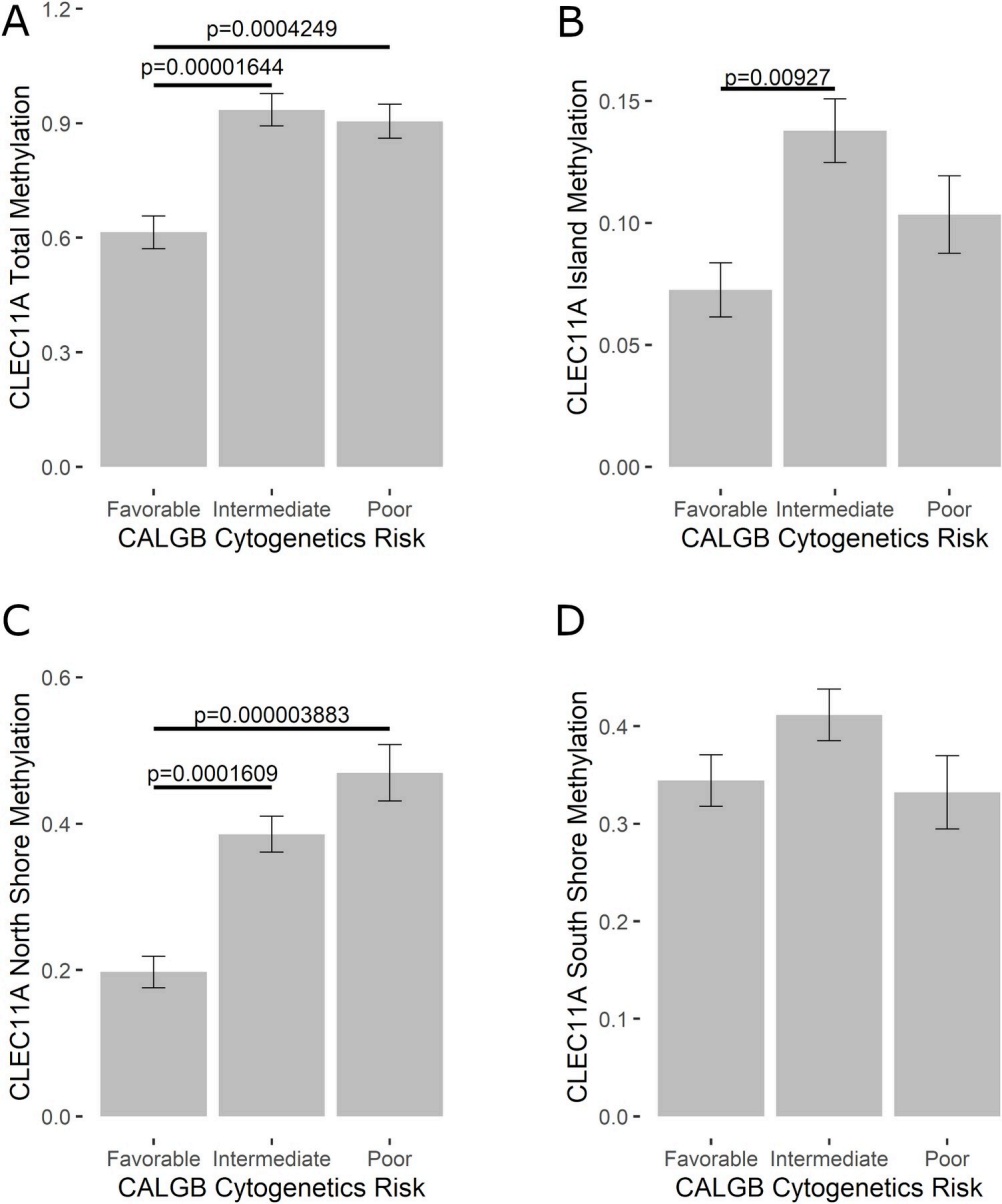
Methylation of *CLEC11A* across cytogenetic risk factor classification. Average methylation of the *CLEC11A* gene was analyzed based on favorable, intermediate, or poor risk prognosis. Total methylation (A; *p* = 0.00005354), as well as methylation at the CpG island (B; *p* = 0.03174), North Shore (C; *p* = 0.00001247), and South Shore (D; *p =* 0.1991) are shown. Statistical significance was determined utilizing a Kruskal-Willis test with Dunn’s multiple comparisons and Bonferroni correction. Statistical significance between individual risk factor groups is indicated on graphs as appropriate.

Because AML survival is based on a variety of factors, we further analyzed *CLEC11A* methylation in relationship to cytogenetic risk factors, based on genetic abnormalities observed in patient tissues and classified as favorable (n = 24), intermediate (n = 70), or poor (n = 27) risk group. Total methylation (North Shore, Island, and South Shore combined) was analyzed as well as methylation at each individual location on the *CLEC11A* promoter. Kruskal Wallis analysis indicated statistically significant results in total methylation (*p* = 0.00005354; Fig 2A), island methylation (*p* = 0.03174; Fig 2B); and methylation at the North Shore (*p* = 0.00001247; Fig 2C). Pair-wise Dunn’s multiple comparisons analysis with Bonferroni correction resulted in additional statistically significant findings within the total methylation, island, and north shore groups (indicated on graphs in Fig 2). Finally, methylation levels at the South Shore *(p =* 0.1991; Fig 2D) of *CLEC11A* showed no significant differences between risk factor groupings.

### No differences in *CLEC11A* methylation levels compared to demographic data

**Fig 3:**
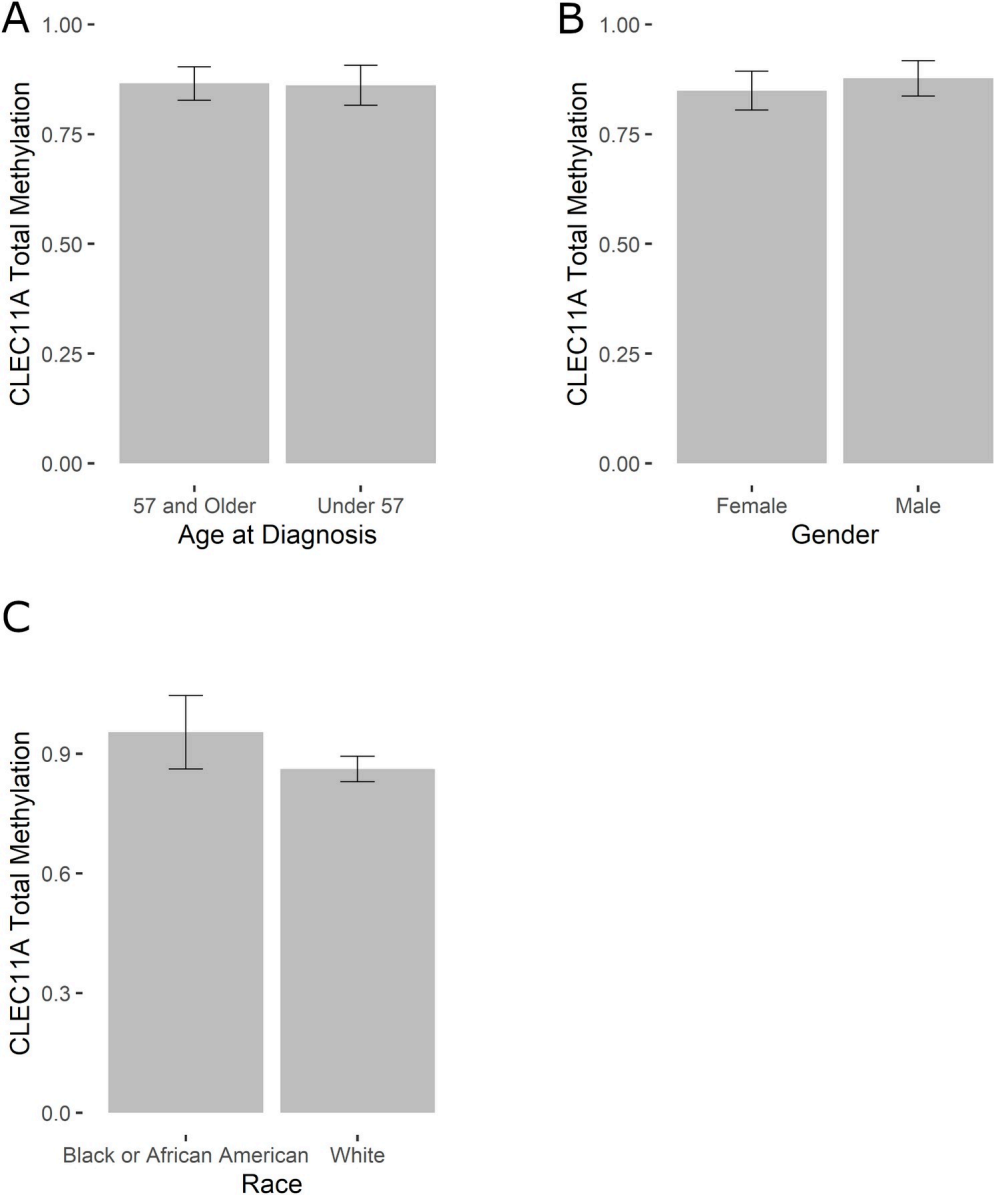
Total *CLEC11A* gene methylation across different demographics. The relative total methylation of the *CLEC11A* gene is shown comparing possible differences between patient age (A; *p* = 0.8277), gender (B; *p =* 0.6707), and race (C; *p =* 0.3032). Statistical significance was determined using a Mann-Whitney Rank Sum test.

To complete our analysis into the factors that affect patient survival, we analyzed the total methylation levels of *CLEC11A* among the same AML patients, based on demographic data of age, gender, and race. When the total methylation for *CLEC11A* was compared between patients ages 57 and older (n = 62) to those under 57 (n = 60), there was no significant difference (*p* = 0.8277; Fig 3A). Furthermore, there was no significant difference in total methylation based on the patient’s gender (*p =* 0.6707; Male n = 63; Female n = 59; Fig 3B) or race (*p =* 0.3032; Black/African American n = 10; White n = 109; Fig 3C). Methylation at the North Shore was also analyzed in all demographic groups with no statistical difference found (age, *p* = 0.568; gender, *p* = 0.7469; race, *p* = 0.9504).

## Discussion

AML is one of the most common types of leukemia in adults and is responsible for over 11,000 deaths each year. While treatments are improving, there is still much that we do not know about the disease and survival rates remain low, especially for older patients. Previous studies have provided conflicting results about the role of *CLEC11A* in cancer [12, 13]. Specifically, Lin and colleagues showed that CLEC11A-expressing lung cancer cells have increased tumor growth in mice and inhibiting *CLEC11A* expression prevented tumor growth [12]. Conversely, Yin and colleagues reported that increased methylation of *CLEC11A*, corresponding to decreased gene and protein expression, led to poor survival in AML patients, indicating worse disease–the opposite of what was seen in lung cancer [13]. Therefore, the goal of this study was to further elucidate *CLEC11A* methylation in AML, paying specific attention to the individual factors that make up patient survival, namely FAB classification, cytogenetic risk factors, age, race, and gender. The results presented here allow us to better understand which specific aspects of patient survival are correlated to *CLEC11A* methylation to allow for better identification, diagnosis, and potential treatment in the clinic.

Our results showed a general trend of high methylation, corresponding to decreased *CLEC11A* expression, in the M0 subtype, and lower methylation levels in other subtypes (Fig 1). However, it is important to note that AML is not staged with regard to tumor progression the way that other cancers are staged, and FAB classification does not necessarily correspond to worse prognosis or survival rates [4]. However, M0 is generally considered a poor prognosis as cells are undifferentiated and difficult to treat. Cytogenetic analysis to determine genetic abnormalities in patients tends to better correlate with survival and our results show highest methylation of *CLEC11A* in patients with intermediate and poor genetic risk factors compared to those with favorable risk factors (Fig 2).

The results shown here expand on the work of Yin and colleagues, which primarily focused on *CLEC11A* expression rather than methylation, although methylation was examined with regard to overall survival. Additionally, when methylation was examined by Yin and colleagues, there was no assessment of methylation at specific loci of *CLEC11A*, which our data show varies significantly between loci. Finally, while Yin et al. completed an initial assessment of clinical and genetic risk factors for *CLEC11A*, the analysis was focused on a division of patient samples into low versus high expression (based on the median) rather than analyzing *CLEC11A* methylation in all patients who fit within a specific demographic. As stated above, survival in AML is based on a variety of factors including classification of the tumor, genetic risk factors, and demographics. Comparing our results to those of Yin and colleagues, it can be observed that we found far more instances of statistical differences between different FAB stages and cytogenetic risk factors by examining methylation at specific loci and including all patients who fit into the staging or cytogenetic category, rather than comparing high to low expression. Taken together, these data indicate that increased methylation of *CLEC11A* leads to poor survival–building on and expanding the work completed by Yin and colleagues [13]–specifically through its link to poor genetic risk factors and FAB classification.

These results also match the known biological function of CLEC11A as a growth factor for primitive hematopoietic cells. High methylation levels generally correlate with low gene and protein expression levels. Therefore, we expect that when we see high methylation of *CLEC11A* that there is very little protein available to do work in the cell, leading to an increase in undifferentiated hematopoietic cells. The fact that we observe the highest methylation levels, corresponding to lowest gene/protein expression, in M0 subtype may indicate a role for CLEC11A in prognosis and treatment of AML since M0 cells are undifferentiated.

While we observed statistically significant differences in *CLEC11A* methylation levels for FAB classifications and genetic risk factors, we did not observe any significant differences between other demographic data including age, gender, and race, even though these factors are known to impact survival in AML (Fig 3). It is possible that this is due to a small sample size of only 122 patients. However, it is also possible that our results emphasize the purely molecular and cellular role of *CLEC11A* which would indicate that it may be used as a molecular marker in the future without regard to other demographic differences.

Finally, it is important to note that Lin and colleagues observed opposing survival results in lung cancer [12]. Namely, they showed that *CLEC11A*-expressing cells promoted tumor formation in mice, with markers for angiogenesis, which we would assume would lead to poor survival. CLEC11A is primarily known for its role in hematopoiesis, while lung epithelia are an entirely different lineage of cells. Therefore, it is possible that CLEC11A is not having a direct effect on lung epithelia and, instead, that the results seen are due to an indirect response to the protein function. It is known that CLEC11A functions through the ERK pathway, which is known to have a significant role in lung tumor progression. Therefore, it is possible that increased expression of ERK, coinciding with increased CLEC11A, leads to the tumor progression seen in lung cancer models. Furthermore, the fact that markers for angiogenesis are observed in *CLEC11A*-expressing mice could imply that CLEC11A is acting in its normal role as a growth factor for primitive hematopoietic cells, leading to increased angiogenesis in non-blood tissues, such as the lung epithelia. If future research determines that this is the case, it will indicate that these seemingly contradictory studies are not necessarily conflicting. Instead, CLEC11A maintains its function to act as a growth factor of hematopoietic cells throughout the body, but that function leads to drastically different results based on the tissue type on which it is acting. Finally, it is important to note that the work performed by Lin and colleagues was pre-clinical in nature, focusing on cell and mouse models [12]. While these models are invaluable in cancer research, it is also known that the results shown in pre-clinical models do not always match what we observe in patients. Therefore, future work should analyze patient data to determine if the CLEC11A findings persist in lung cancer patients and if changes in methylation are observed.

Taken together, the results shown here indicate a molecular role for methylated *CLEC11A* in prognosis of AML patients. Future studies may wish to examine methylation of *CLEC11A* in additional leukemia types to determine if the results are consistent and potentially expand to other cancer types with a goal to develop better diagnostics and treatments for cancer patients.
